# Tonsils, Adenoids and the Teeth

**Published:** 1920-11

**Authors:** George W. Goler

**Affiliations:** Health Officer of the City of Rochester


### TONSILS, ADENOIDS AND THE TEETH

BY GEORGE W. GOLER, M.D.
*Health Officer of the City of Rochester*

In that not so very far-away time when everybody was sick, we thought the greater part of the way of life was the way of sickness. Now we know that the larger way of life is health instead of sickness, and we are learning that the best way to care for the health of people is not to cure them of their sickness, but to prevent sickness, and so we are preventing smallpox, whooping cough, diphtheria, typhoid, and many other diseases. We are caring for the teeth of our children so that when they grow up they may not suffer from joint infection or what is called rheumatism, so they will be able to chew their food properly, digest it easily and thus become physically and mentally stronger.

We, too, have learned that good sets of teeth provoke smiles and laughter, and that mirth and joy are largely aids to health.

These things we have discovered, and we are making other discoveries. We have found that in the back part of the throat there are certain glands known as tonsils and adenoids. These glands are what are known as accessory salivary glands and in early infancy probably render useful service to the body. But they, like other glands, the thymus for instance, too often grow instead of shrinking, and when they enlarge they obstruct the nose and throat and furnish many little nests in which dangerous kinds of bacteria may grow, multiply and interfere with the growth, development and health of the body by poisoning it. When these tonsils and adenoids persist they interfere with the growth and alter the shape of the face and cause the jaw to recede, the upper lip to protrude, the "hatchetshaped'' face, enlarged glands in the neck, distortions of the teeth in their settings, earache, and decay of teeth.

But tonsils and adenoids do other things. Children so affected are more likely to contract infectious diseases, such as whooping cough, which may lay the foundation for consumption; measles, which causes nearly 10 per cent. of the deafness in later life; scarlet fever, which causes much of the heart and kidney disease and many of the apoplexies after 60, and the joint affections or so-called rheumatism. These, all these, and other diseases, we may do much to avoid by removing infected tonsils and adenoids.

Now these tonsils and adenoids when infected, need not grow very large to cause trouble of this kind. Sometimes when they shrink they cause just as much damage as when they are large, and the small and the large tonsil should be removed very early. Remember, children with infected tonsils and adenoids more easily contract infectious diseases.

One of the difficulties that we have to contend with in our attempts to promote health, is that the promotion of health is very much delayed. We wait until the growth, the nutrition, the tonsil obstruction, enlarged glands, tooth decay, earache have ineffaceably stamped themselves upon the faces and health of our children before we do anything to protect them. We know we ought to care for the health of our children. We know how to do it. We have had all this knowledge for many years. We have been asking ourselves why we don't take advantage of the information we have. Why?

Through the tonsil-adenoid clinic conducted by the Rochester Dental Dispensary the way has been opened for us to make of Rochester children, our children, your children, the very best physical children in the world. If we do what we ought to do, what an example could Rochester set before the world! Will we do it? Will we rid our children of tonsils and adenoids? Of course we will!

More than 1,400 of these children have been taken to the Rochester Dental Dispensary where their tonsils and adenoids have been removed, and these children, all of them, serve as living examples to the thousands of other children who need such operations. What a good thing it is to keep children well, to seize early the opportunity of keeping them well, so they may get the full benefits of that education offered here in the Rochester schools. So many of these children with infected tonsils and adenoids are backward because they can not breathe, because they are being poisoned by the excretions of their tonsils and adenoids. A child who can't breathe can't digest, and such children can't grow, can't learn, can't properly prepare themselves for manhood and womanhood; can't be prepared to earn all they deserve to earn in the future struggle for existence.

Now, at the Rochester Dental Dispensary clinic, which I regard as one of the greatest experiments in public health that has ever taken place in a short space of time in Rochester, we have had more than 1,400 children operated upon for the removal of tonsils and adenoids. There are several other thousands who need the same operation. They need it early. Will you not give your children the benefit of at least an examination to determine whether they need this operation or not? For your sake, for the children's sake—for their future health, growth, development, education, earning power—tonsil-adenoid removal should not be neglected.

